# Predicting the clinical course of advanced systemic mastocytosis on treatment with avapritinib or midostaurin

**DOI:** 10.1038/s41375-026-03149-0

**Published:** 2026-10-09

**Authors:** Johannes Lübke, Jason Gotlib, Daniel J. DeAngelo, Juliana Schwaab, Deepti H. Radia, Michael Deininger, Tracy I. George, Ilda Bidollari, Saša Dimitrijević, Javier I. Muñoz-González, Hongliang Shi, Andreas Reiter

**Affiliations:** 1 Department of Hematology and Oncology, University Hospital Mannheim, Heidelberg University, Mannheim, Germany; 2 Stanford University School of Medicine and Stanford Cancer Institute, Stanford, CA USA; 3 Department of Medical Oncology, Dana-Farber Cancer Institute, Boston, MA USA; 4 Guy’s & St Thomas’ NHS Foundation Trust, London, UK; 5 Division of Hematology/Oncology, University of Michigan, Ann Arbor, MI USA; 6 ARUP Laboratories, University of Utah, Salt Lake City, UT USA; 7 Blueprint Medicines Corporation, Cambridge, MA USA

**Keywords:** Myeloproliferative disease, Haematological cancer

## Abstract

Advanced systemic mastocytosis (AdvSM) is driven by *KIT* D816V in approximately 95% of cases. Accurate assessment and prediction of clinical course and disease outcome before and during treatment with the KIT inhibitors midostaurin and avapritinib may facilitate optimization of treatment strategies. We retrospectively evaluated clinical, morphologic, laboratory, and genetic predictors of outcome in 79 midostaurin- and 176 avapritinib-treated patients. The number of mutations at baseline in *SRSF2*/*ASXL1*/*EZH2* (midostaurin) and in *SRSF2/RUNX1/SETBP1* (avapritinib) delineated highly predictive 3-tier risk classification systems. Treatment with avapritinib was associated with an early response of the *KIT* D816V variant allele frequency; a ≥10% reduction in peripheral blood at day 15 indicated improved overall survival (OS). Within 3 months of starting treatment, a ≥25% increase in alkaline phosphatase (AP) levels (AP flare) was observed in 46/47% of midostaurin/avapritinib-treated patients and was associated with longer OS. Multivariable analyses identified a ≥25% reduction in the *KIT* D816V allele burden (midostaurin), normalization of serum albumin levels (avapritinib), and normalization of hemoglobin and/or platelet levels (midostaurin and avapritinib) within 6 months as significant predictors for prolonged OS. In conclusion, genetic profiling, dynamic laboratory parameters, and *KIT* D816V monitoring improve outcome prediction in patients with AdvSM on midostaurin or avapritinib.

## Introduction

Systemic mastocytosis (SM) is a myeloid neoplasm characterized by multifocal accumulation of neoplastic mast cells (MCs) predominantly in the bone marrow (BM) and visceral organs. According to the 2022 revised World Health Organization classification of hematolymphoid tumors (WHO-HAEM 5) and International Consensus Classification (ICC), advanced SM (AdvSM) comprises aggressive SM (ASM), SM with an associated hematologic (WHO-HAEM 5)/myeloid (ICC) neoplasm (SM-AHN/SM-AMN), and mast cell leukemia (MCL) [1–3].

The genetic landscape of SM has been intensively studied [4–15]. Approximately 95% of patients with AdvSM harbor the canonical driver mutation *KIT* D816V, which arises in early hematopoietic stem and progenitor cells, leading to multilineage involvement [16]. Approximately 70% of patients with AdvSM harbor additional somatic mutations, most frequently *TET2*, *SRSF2*, *ASXL1*, and *RUNX1* [4, 9, 10]. The combination of selected high-risk mutations (e.g., *SRSF2, ASXL1, RUNX1*) with other clinical characteristics has led to the development of several risk scoring systems [17–20].

The primary treatment approach is to reduce mast cell burden, alleviate end-organ damage, and extend overall survival (OS) in AdvSM. In the past, this relied on cytoreductive therapy involving the purine analog cladribine [20–26] and, where feasible, allogeneic hematopoietic cell transplantation (alloHCT) [27–29]. The advent of targeted treatment options such as midostaurin, a multikinase inhibitor targeting D816V-mutated and wild-type KIT, PDGFRA/B, VEGFR2, and FLT3 [20, 30–37] and avapritinib, a potent inhibitor of D816V-mutated KIT [21, 38–41], has transformed AdvSM management.

Techniques to assess treatment response include the measurement of C-findings (MC-related organ damage), serum tryptase levels, BM MC infiltration, and *KIT* D816V variant allele frequency (VAF). For a better understanding of potential sequential treatment strategies for AdvSM, reliable and robust markers of clinical response, drug resistance, and disease progression on midostaurin and avapritinib are needed. We therefore evaluated baseline and on-treatment parameters in patients with AdvSM receiving midostaurin and/or avapritinib.

## Patients and methods

### Study population

The avapritinib cohort comprised 176 patients with AdvSM (ASM [*n* = 29, 16%], SM-AHN [*n* = 119, 68%], MCL ± AHN [*n* = 28, 16%]) from the phase 1 EXPLORER (NCT02561988) [40] and phase 2 PATHFINDER (NCT03580655) [41–43] clinical trials. Treatment with avapritinib was initiated between 2016 and 2021 (overall follow-up 398 patient-years). Prior treatment with midostaurin or cladribine was seen in 81 patients (46%) and 22 patients (13%), respectively. Progression to secondary acute myeloid leukemia (AML) occurred in eight patients (5%). In EXPLORER, the starting dose of avapritinib was escalated from 30 to 400 mg once daily, while in PATHFINDER, all but two patients received a starting dose of 200 mg once daily.

The midostaurin cohort comprised 79 patients with AdvSM (ASM [*n* = 11, 14%], SM-AHN [*n* = 50, 63%], MCL ± AHN [*n* = 18, 23%]) from the German Registry on Eosinophils and Mast Cells (GREM). Treatment with midostaurin, at a starting dose of 100 mg twice daily in 63 (80%) patients, was initiated between 2009 and 2021 (overall follow-up 239 patient-years). Prior treatment with cladribine was seen in 16 (20%) patients. Nine patients subsequently received avapritinib in the second-line setting over an accumulated follow-up period of 10.2 patient-years from the start of midostaurin therapy. Progression to secondary MCL and secondary AML occurred in 10 (13%) and seven (9%) patients, respectively.

Clinical, laboratory, and molecular parameters were obtained at the time of diagnosis and/or first referral to the treatment center, at treatment initiation with midostaurin or avapritinib (Table 1), and at multiple time points during treatment. Per protocol, patients were treated until disease progression and/or unacceptable toxicity, as adjudicated by the treating physician.

**Table 1:** Baseline characteristics.

	Midostaurin	Avapritinib
Number of patients, *n* (%)	79 (100)	176 (100)
Age in years at treatment initiation, median (range)	67 (28–87)	68 (31–88)
Male, *n* (%)	48 (61)	103 (59)
Diagnosis		
ASM, *n* (%)	11 (14)	29 (16)
SM-AHN, *n* (%)	50 (63)	119 (68)
MCL ± AHN, *n* (%)	18 (23)	28 (16)
AHN		
CMML, *n* (%)	21 (27)	57 (32)
MDS/MPN, *n* (%)	24 (30)	31 (18)
MDS, *n* (%)	5 (6)	18 (10)
CEL, *n* (%)	6 (8)	7 (4)
Others, *n* (%)	7 (9)	6 (3)
Relevant laboratory and clinical parameters for the assessment of C-findings		
Hemoglobin, g/dL, median (range)	10 (7–15)	11 (7–17)
<10 g/dL, *n* (%)	34 (43)	63 (36)
Platelets, × 10^9^/L, median (range)	110 (7–1 253)	150 (28–992)
<100 × 10^9^/L, *n* (%)	34 (43)	60 (34)
ANC, × 10^9^/L, median (range)	5 (1–67)	5 (0–44)
<1 × 10^9^/L, *n* (%)	3 (4)	8 (5)
AP, U/L, median (range)	198 (41–1 162)	155 (27–1 747)
>150 U/L, *n* (%)	52 (66)	92 (52)
Albumin level, g/L, median (range)	35 (25–43)	40 (13–50)
<34 g/L, *n* (%)	34 (43)	33 (19)
Weight loss (>10% over 6 months), *n* (%)	56 (71)	38 (22)
Other relevant parameters		
Leukocytes, × 10^9^/L, median (range)	9 (0–131)	9 (1–45)
Monocytes, × 10^9^/L, median (range)	1 (0–7)	1 (0–10)
Eosinophils, × 10^9^/L, median (range)	0 (0–13)	0 (0–24)
MC infiltration in BM, %, median (range)	50 (1–100)	40 (1–95)
Serum tryptase level, µg/L, median (range)	189 (12–1 690)	216 (12–1 600)
*KIT* D816V EAB/VAF in PB, %, median (range)	25 (0–74)	15 (0–80)
MARS at diagnosis		
Low risk, *n* (%)	21 (27)	65 (37)
Intermediate risk, *n* (%)	20 (25)	37 (21)
High risk, *n* (%)	38 (48)	74 (42)
Prior treatment		
Cladribine, *n* (%)	16 (20)	22 (13)
Midostaurin, *n* (%)	—	81 (46)

*AHN* associated hematologic neoplasm, *ANC* absolute neutrophil count, *AP* alkaline phosphatase, *ASM* aggressive systemic mastocytosis, *BM* bone marrow, *CEL* chronic eosinophilic leukemia, *CMML* chronic myelomonocytic leukemia, *EAB* expressed allele burden, *MARS* mutation-adjusted risk score, *MC* mast cell, *MCL* ± *AHN* mast cell leukemia with/without an associated hematologic neoplasm, *MDS* myelodysplastic syndrome, *MDS/MPN* myelodysplastic syndrome/myeloproliferative neoplasm, *PB* peripheral blood, *SM-AHN* systemic mastocytosis with an associated hematologic neoplasm, *VAF* variant allele frequency.

### Ethics approval and consent to participate

The study design adhered to the principles of the Declaration of Helsinki and was approved by the relevant institutional review boards. All data reported in this paper were derived from samples previously obtained as part of the EXPLORER, PATHFINDER, and GREM studies. The protocols for PATHFINDER and EXPLORER were approved by the Institutional Review Board or Independent Ethics Committee at each participating center. The analysis of patients from the GREM cohort was approved by the Ethics Committee of the Medical Faculty Mannheim, Heidelberg University, Germany (2016-506N-MA, 2014-617N-MA). No new patient interventions were performed as part of this study. All patients provided written informed consent.

### Gene mutation analyses

For the midostaurin-treated patients, quantitative assessments of the *KIT* D816V expressed allele burden (EAB) were performed using allele-specific quantitative real-time reverse-transcriptase polymerase chain reaction analysis on RNA/complementary DNA (limit of detection: 0.1%). For the avapritinib-treated patients, quantitative assessments of the *KIT* D816V VAF were performed using droplet digital polymerase chain reaction on DNA (limits of detection: 0.17% in EXPLORER and 0.022% in PATHFINDER).

Next-generation sequencing analyses of DNA derived from peripheral blood (PB) samples (MiSeq Instrument [Illumina, San Diego, CA]) were used to identify variants of genes frequently altered in myeloid neoplasms. The sequencing panel consisted of 54 genes in the avapritinib group and 33 recurrently mutated genes in myeloid neoplasms in the midostaurin group. The mutation tier grading was assigned based on the ClinVar database, OncoKB database, Single Nucleotide Polymorphism database, and Genome Aggregation database. Tier 1 was assigned to pathogenic and likely pathogenic variants for both drugs; in addition, Tier 2 was assigned to variants of unknown significance for avapritinib. Tier 3 variants assigned to benign and likely benign variants were excluded from the study.

### Statistical analyses

The Mann–Whitney U-test was used to compare continuous variables and medians of distributions. Fisher’s exact test was used for categorical variables. We retrospectively analyzed the OS (defined as time from treatment initiation to the date of last follow-up [if alive] or date of death) using the Kaplan–Meier method with the log-rank test for group comparisons/visualizations. The duration of treatment (DOT) represents the interval from the start of midostaurin or avapritinib to its discontinuation. For the estimation of hazard ratios (HRs) and multivariable analysis, the Cox proportional hazards regression model was used. All variables that showed prognostic significance in univariate analyses were included in the multivariable analysis. Continuous variables with at least 5 observations in any given category were considered for prediction of OS. The cut-offs for on-treatment parameters were defined using a minimum *p* value approach, selecting the threshold that maximized OS separation by log-rank testing. *P* values of <0.05 (2-sided) were significant.

## Results

### Treatment modalities, DOT, and OS

In the pooled population from EXPLORER and PATHFINDER, avapritinib was used in first line (1L) or second and further lines (2L+) in 66 (38%) and 110 (62%) patients, respectively. A median of 1 prior line was administered (range, 0–6). On avapritinib 1L, the median time from diagnosis to treatment and DOT with avapritinib were 0.3 months and 22.9 months, respectively. On 2L+, median DOT was 22.3 months. Median OS of the overall cohort was not reached (95% CI, 49.0–not reached [NR]).

In the midostaurin group, 45 (57%) and 34 (43%) patients received midostaurin in 1L or 2L+, respectively, after a median of 1 prior treatment line (range, 0–4). Median time from diagnosis to treatment and DOT with midostaurin in 1L were 10.1 months (95% CI, 7.2–12.0) and 11.6 months (95% CI, 7.6–17.1), respectively. In 2L+, median DOT was 13.9 months (95% CI, 3.5–30.5). Median OS of the overall cohort was 36.8 months (95% CI, 23.5–50.1).

### Prognostic impact of additional somatic mutations at baseline

**Fig. 1:**
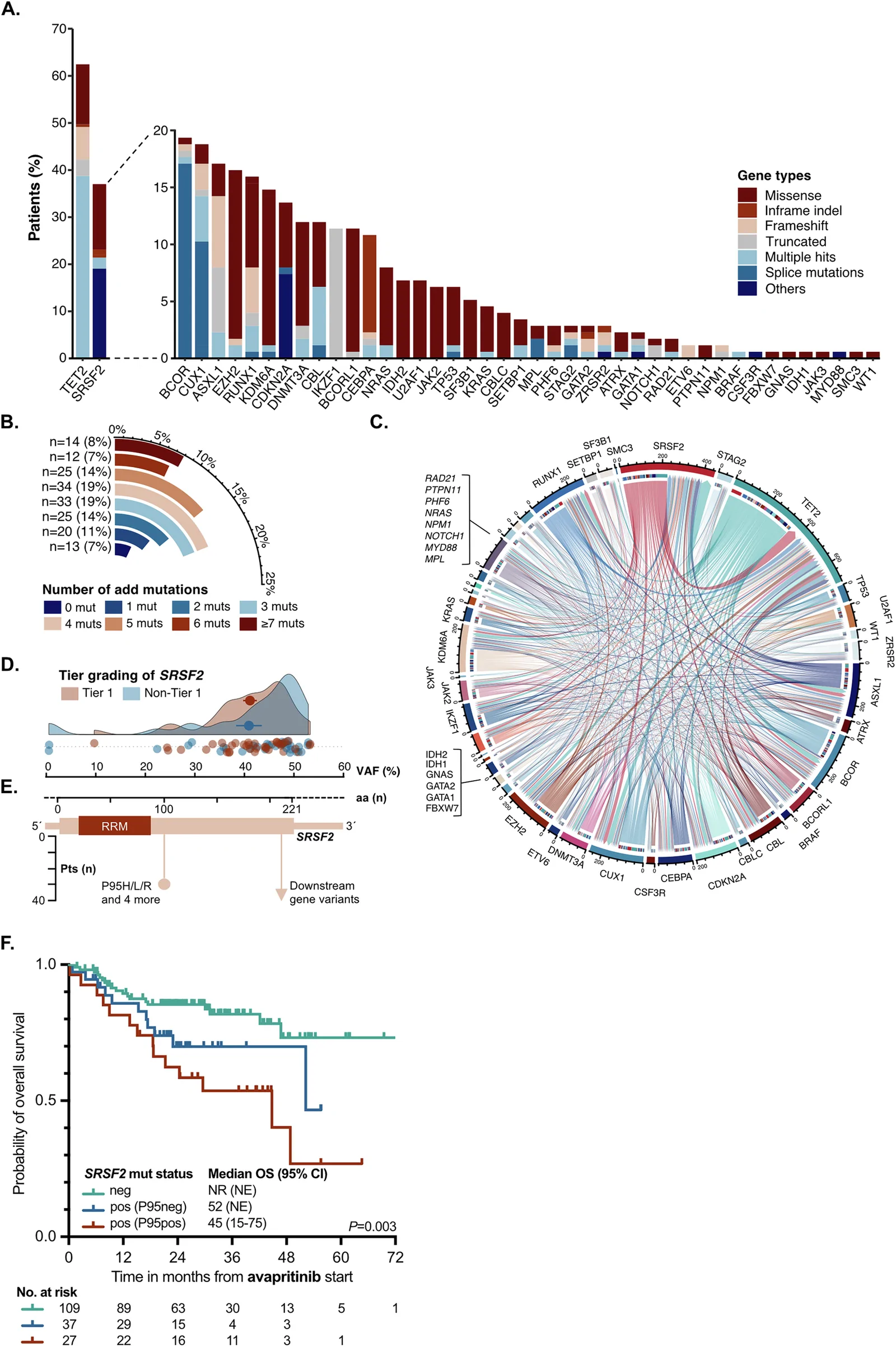
Genomic landscape of additional somatic mutations beyond the canonical driver mutation *KIT* D816V (and other rare D816 variants). **A** shows the frequency of mutations in 176 patients with AdvSM treated with avapritinib. Mutations were categorized by type (missense, in-frame deletions or insertions [in-frame indel], frameshift deletions or insertions, and whether they resulted in a truncation or affected a splice site). Colors represent the mutation type. **B** presents the frequency of additional somatic mutations beyond *KIT*. In **C** the Circos diagram highlights the intricate molecular background of AdvSM. The links between different genetic lesions are represented using various colors, with directional arrows indicating their relationships. The width of the links reflects the strength of these relationships. The sector widths signify the overall strength of a genetic lesion to connect or be connected to other genetic lesions. **D** shows the VAF distribution in *SRSF2* depending on a predefined tier grading. *SRSF2* P95 mutations were considered Tier 1, while mutations at other locations were considered non-Tier 1. For more information on the tier grading, please see the Methods section. **E** depicts the location of mutations within and outside the *SRSF2* gene and their protein consequence. **F** displays Kaplan–Meier probability estimates of OS in avapritinib-treated patients with AdvSM, stratified by the presence/absence and specific type of *SRSF2* mutations. OS data were available in 173 patients. Pairwise log-rank comparisons yielded the following results: “neg” vs. “pos (P95neg),” *p* = 0.069; “pos (P95neg)” vs. “pos (P95pos),” *p* = 0.235. Abbreviations**:** *aa* amino acids, *add* additional, *AdvSM* advanced systemic mastocytosis, *CI* confidence interval, *mut* mutation, *NE* not evaluable, *neg* negative, *NR* not reached, *OS* overall survival, *pos* positive, *pts* patients, *RRM* RNA recognition motif, *VAF* variant allele frequency.

The total number of additional somatic mutations and the relative frequencies of individual genes were comparable between avapritinib- and midostaurin-treated cohorts when considering only Tier 1 mutations (Fig. 1 and Supplementary Figs. S1 and S2). A comparison of VAFs between Tier 1 and Tier 2 additional somatic mutations is shown in Fig. 1D and Supplementary Fig. S3.

In the avapritinib cohort, 167/176 (95%) patients were *KIT* D816V positive. Overall, 163 (93%) patients had at least one additional somatic mutation in varied combinations (median, 4; range, 1–20), most frequently *TET2* (*n* = 108, 61%) and *SRSF2* (*n* = 64, 36%). In total, 27/64 (42%) and 37/64 (58%) either carried an *SRSF2* hotspot mutation at p95 or variable mutations downstream of p95. While different p95 hotspot mutations (P95H, P95L, P95R, P95RP, P95T) did not translate into distinct survival outcomes (*p* = 0.985; Supplementary Fig. S4), the median OS significantly differed between patients with a p95 hotspot mutation (45 months [95% CI, 14.7–75.2]), an *SRSF2* downstream gene variant (52 months [95% CI, not evaluable (NE)–NE]), and patients without any *SRSF2* mutation (NR [95% CI, NE–NE]; *p* = 0.003; Fig. 1).

In the midostaurin cohort, 74/79 (94%) patients were *KIT* D816V-positive, and 59 (75%) had at least one additional somatic mutation (median, 1; range, 1–5), most frequently *TET2* (*n* = 36, 46%), *SRSF2* (*n* = 36, 46%), and *ASXL1* (*n* = 15, 19%). Mutations in *EZH2* and *JAK2* tended to be mutually exclusive (Supplementary Fig. S2).

**Fig. 2:**
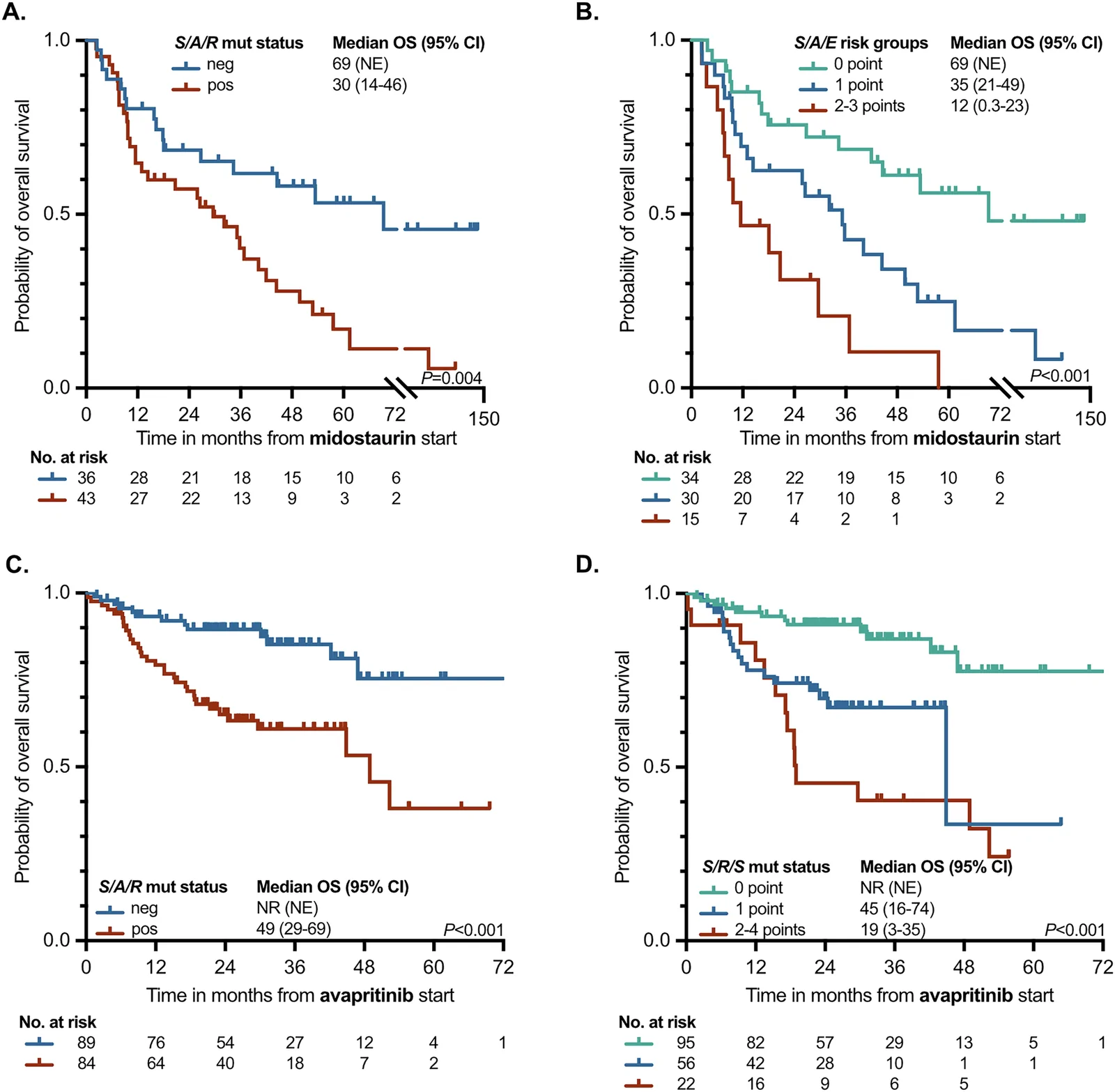
The analysis of a baseline mutational profile allows risk stratification in midostaurin- and avapritinib-treated patients with AdvSM. Kaplan–Meier probability estimates of OS in midostaurin-treated patients with AdvSM based on the presence/absence of mutations within the mutational panel *S/A/R* (**A**) and on a hazard-weighted risk scoring system (**B**; *EZH2*, 2 points; *SRSF2*, 1 point; *ASXL1*, 1 point) are shown (data available in 79 patients). Pairwise log-rank comparisons yielded the following results: “0 point” vs. “1 point,” *p* = 0.008; “1 point” vs. “2–3 points,” *p* = 0.034. Kaplan–Meier probability estimates of OS in avapritinib-treated patients with AdvSM, stratified by the presence/absence of mutations within the mutational panel *S/A/R* (**C**) and on a hazard-weighted risk scoring system (**D**; *SETBP1*, 2 points; *RUNX1*, 1 point; *SRSF2*, 1 point) are shown (data available in 173 patients). Pairwise log-rank comparisons yielded the following results: “0 point” vs. “1 point,” *p* < 0.001; “1 point” vs. “2–4 points,” *p* = 0.116. Abbreviations: *AdvSM* advanced systemic mastocytosis, *CI* confidence interval, *mut* mutated, *NE* not evaluable, *neg* negative, *NR* not reached, *OS* overall survival, *pos* positive, *S/A/R SRSF2*/*ASXL1*/*RUNX1*.

Based on the presence or absence of the established high-risk mutations in *SRSF2*/*ASXL1*/*RUNX1 (S/A/R)*, median OS differed significantly in avapritinib- and midostaurin-treated patients and ranged from NR and 69 months (absence of any mutation) to 49 months and 30 months (presence of at least 1 mutation), respectively (*p* < 0.001; *p* = 0.004; Fig. 2A and C).

**Fig. 3:**
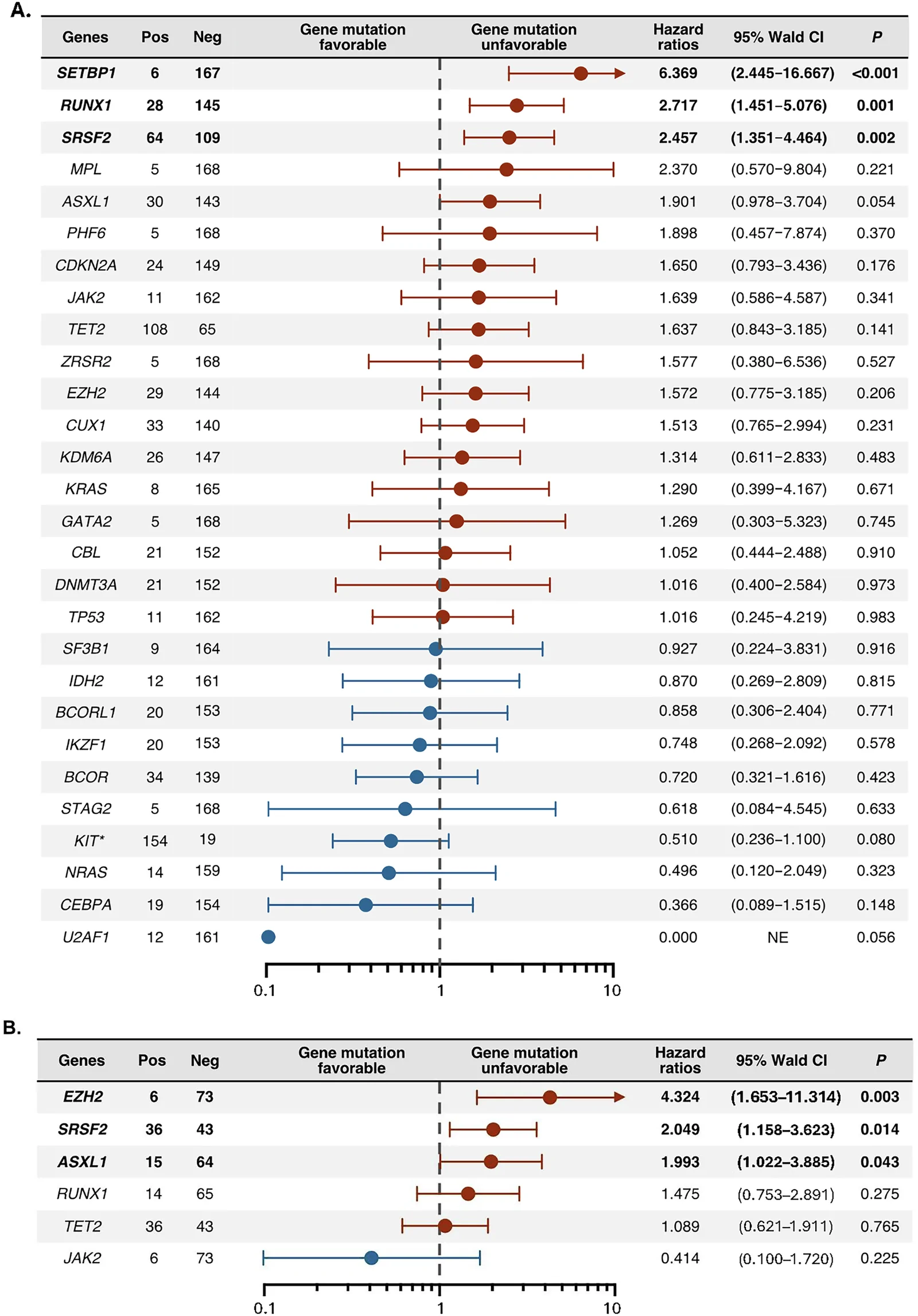
Genomic correlates of overall survival in patients with AdvSM. The analysis encompassed only gene mutations present in a minimum of five patients. For the univariate analyses, all Tier 1 and Tier 2 mutations within a single gene were considered. Significant results are highlighted in bold. **A** encompasses data on avapritinib treatment (data available in 173 patients). When focusing solely on Tier 1 mutations, significant HRs were only seen for *SRSF2* (2.309 [1.222–4.367], *p* = 0.008) and *SETBP1* (5.618 [1.965–16.129], *p* < 0.001) mutations. Significant results are highlighted in bold. *All *KIT* mutations were considered. When specifically focusing on *KIT* D816V, the HR was 0.534 (95% CI, 0.274–1.041, *p* = 0.060). Among patients with mast cell leukemia, the HR for the presence of any *KIT* mutation was 0.109 (95% CI, 0.022–0.543, *p* = 0.007), and for *KIT* D816V specifically, 0.110 (95% CI, 0.020–0.604, *p* = 0.011). **B** summarizes data on midostaurin (data available in 79 patients). Four patients were *KIT* D816V negative. Abbreviations: *AdvSM* advanced systemic mastocytosis, *CI* confidence interval, *NE* not evaluable, *neg* negative, *pos* positive.

In the avapritinib cohort, the presence of additional somatic mutations in *SETBP1* (HR 6.369 [95% CI, 2.445–16.667], 2 points), *SRSF2* (HR 2.457 [1.351–4.464], 1 point), and *RUNX1* (HR 2.717 [95% CI, 1.451–5.076], 1 point; Fig. 3A) delineated a highly predictive, hazard ratio-weighted 3-tier risk categorization (0 points, *n* = 95, 55%, median OS NR; 1 point, *n* = 56, 32%, 45 months; 2–4 points, *n* = 22, 13%, 19 months; *p* < 0.001; Fig. 2D). Mutations in *ASXL1* (HR 1.901 [0.978–3.704]) were only associated with a numerically shorter OS (Fig. 3A). In patients with MCL, *KIT* D816V mutations were associated with a favorable prognosis (HR 0.110 [95% CI, 0.020–0.604]).

In the midostaurin cohort, a hazard ratio-weighted 3-tier risk categorization based on mutations in *EZH2* (HR 4.3 [95% CI, 1.7–11.3], 2 points), *SRSF2* (HR 2.0 [95% CI, 1.2–3.6], 1 point), and *ASXL1* (HR 2.0 [95% CI, 1.0–3.9], 1 point; Fig. 3B) significantly stratified 3 different cohorts (0 points, *n* = 34, 43%, median OS 69 months; 1 point, *n* = 30, 38%, 35 months; 2–4 points, *n* = 15, 19%, 12 months; *p* < 0.001; Fig. 2B). Mutations in *JAK2* were associated with a trend toward a better prognosis (HR 0.4 [0.1–1.7]; Fig. 3B).

### Prognostic impact of on-treatment parameters

**Fig. 4:**
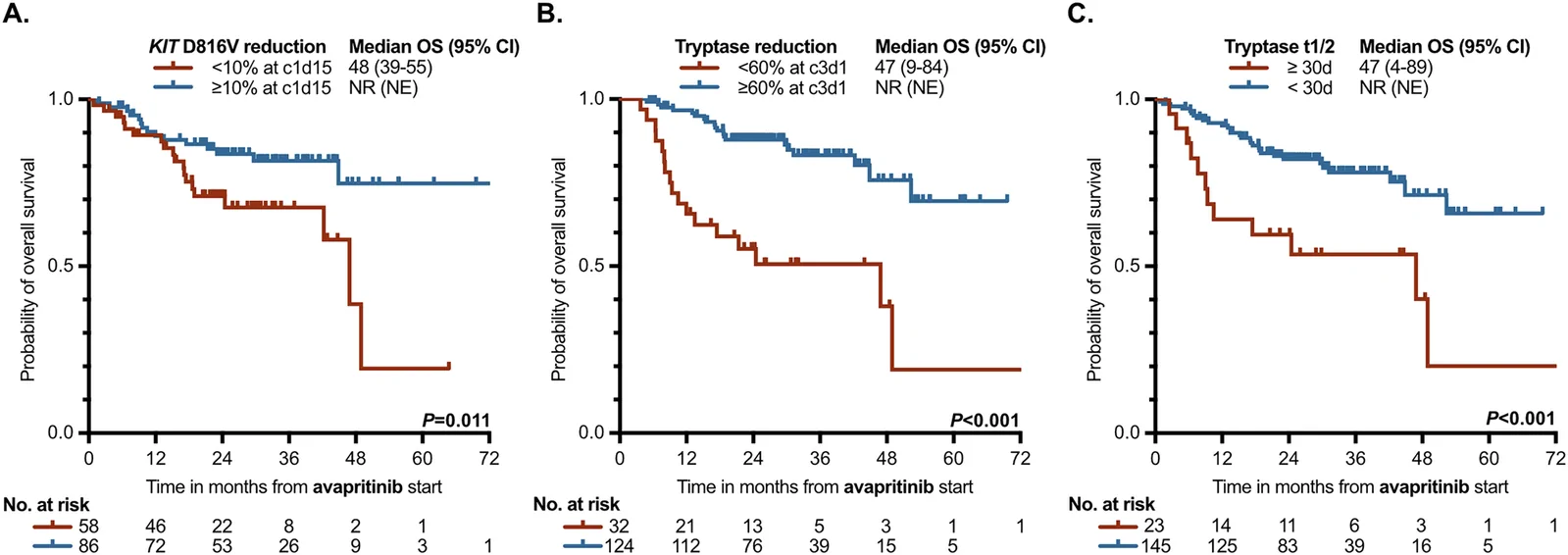
Sequential monitoring of *KIT* D816V and serum tryptase in the peripheral blood allows an early response assessment in avapritinib-treated patients with AdvSM. Kaplan–Meier probability estimates of OS based on *KIT* D816V reduction (**A**; data available in 144 patients), serum tryptase reduction (**B**; data available in 156 patients), and time to 50% reduction in serum tryptase (**C**; data available in 168 patients). Abbreviations: *AdvSM* advanced systemic mastocytosis, *c1d15* Cycle 1 Day 15, *c3d1* Cycle 3 Day 1, *CI* confidence interval, *NE* not evaluable, *NR* not reached, *OS* overall survival.

*Early and rapid response of serum tryptase and KIT D816V VAF to avapritinib*. The median reduction of *KIT* D816V VAF and serum tryptase levels in PB was –12.8% and –70.0%, respectively, at day 15; –42.8% and –79.1%, respectively, at 1 month; and –62.8% and –84.5%, respectively, at 2 months. A ≥10% reduction of *KIT* D816V VAF at day 15 was associated with a more favorable median OS (NR vs. 47 months, *p* = 0.011; Fig. 4A). The median time to the reduction of serum tryptase levels ≥50% was 15 days (range, 5–624 days). A reduction in serum tryptase levels by ≥50% in ≤30 days or by ≥60% at month 3 was predictive of improved OS (NR vs. 47 months, *p* < 0.001 [Fig. 4B] and NR vs. 47 months, *p* < 0.001 [Fig. 4C], respectively). Baseline characteristics of the respective cohorts are summarized in Supplementary Tables S1–S3. Respective analyses for the midostaurin cohort could not be performed due to the retrospective nature of data collection.

**Fig. 5:**
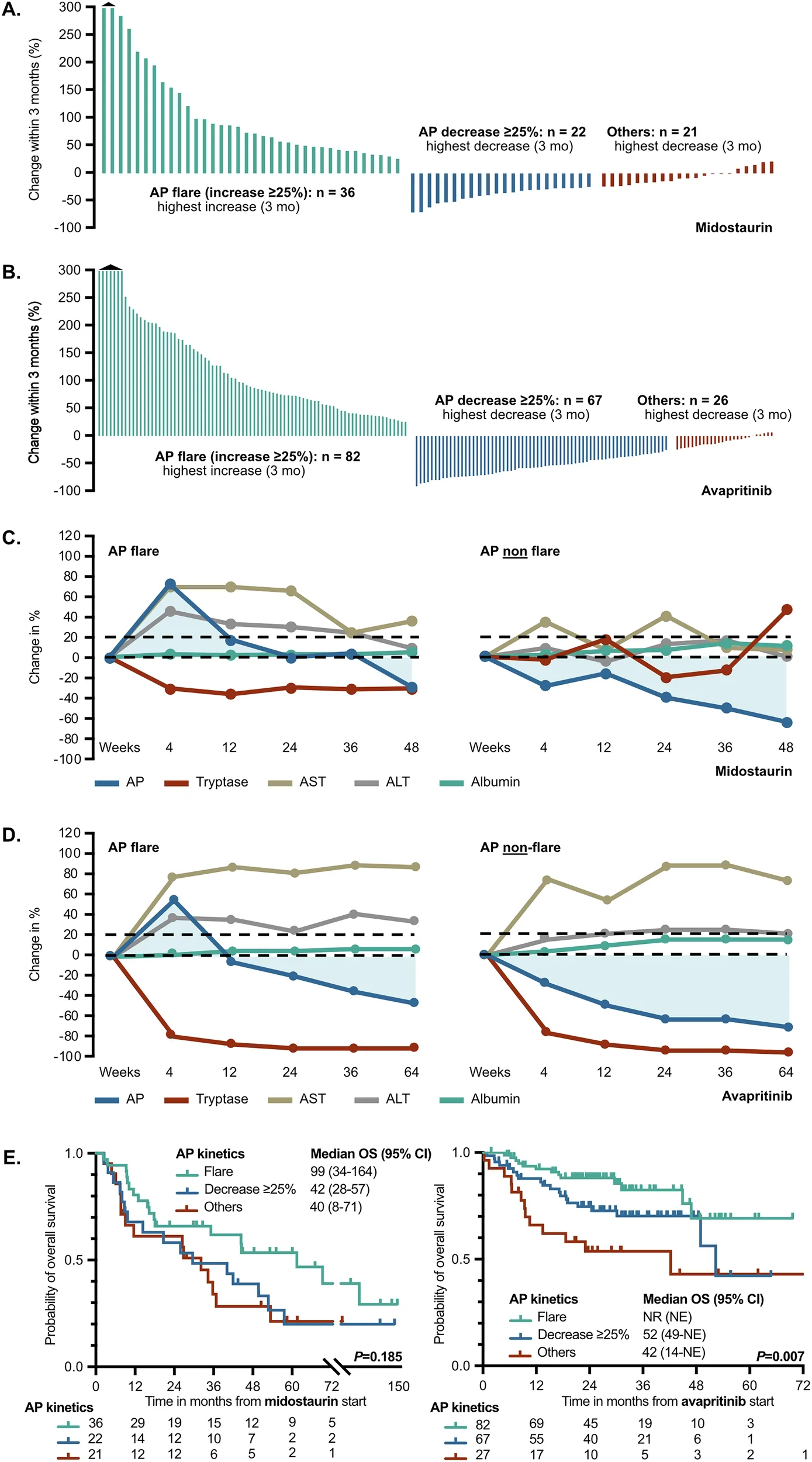
The kinetics of AP levels under midostaurin or avapritinib treatment are predictive for OS in patients with AdvSM. **A**, **B** show the maximal AP change (maximal increase for “AP flare,” maximal decrease for “AP decrease ≥25%,” and maximal decrease/minimal increase for “Others”) within 3 months after treatment start. **C**, **D** depict the profile of AP and additional serum chemistry parameters (serum tryptase, AST, ALT) over time under midostaurin or avapritinib treatment. In **E**, Kaplan–Meier probability estimates of OS in midostaurin- (data available in 79 patients) and avapritinib- (data available in 176 patients) treated patients with AdvSM based on AP kinetics are shown. For midostaurin, pairwise log-rank comparisons yielded the following results: “AP flare” vs. “Decrease ≥25%,” *p* = 0.125; “Decrease ≥25%” vs. “Others,” *p* = 0.976. For avapritinib, pairwise log-rank comparisons yielded: “AP flare” vs. “Decrease ≥25%,” *p* < 0.001; “Decrease ≥25%” vs. “Others,” *p* = 0.047. Abbreviations: *AdvSM* advanced systemic mastocytosis, *ALT* alanine transaminase, *AP* alkaline phosphatase, *AST* aspartate aminotransferase, *CI* confidence interval, *mo* months, *OS* overall survival.

*Alkaline phosphatase (AP) flare*. Within 3 months after start of avapritinib or midostaurin, 82 patients (47%) and 36 patients (46%) had an early increase in serum AP levels ≥25%, respectively (AP flare; range, 26–563%, 26–331%; Fig. 5A and B). Within 6 months after initiating avapritinib or midostaurin, a subsequent drop (range, –1.4 to –86.9%; –3.2 to –82.2%) below baseline was seen in most patients (*n* = 58, 71%, normalization: *n* = 43, 52%; *n* = 19, 53%, normalization: *n* = 19, 53%). In both cohorts, patients with an early AP flare had significantly lower AP values at baseline vs. those without an early AP flare (avapritinib, 134.5 vs. 228.0 U/L, *p* = 0.002; midostaurin, 158 vs. 286 U/L; *p* < 0.001). In midostaurin-treated patients, the AP flare was associated with a median decrease of serum tryptase levels of –37% (range, –79 to 285%) at month 3 (Fig. 5C). In avapritinib-treated patients, serum tryptase levels decreased independently of AP kinetics (median reduction, 90% for patients with and without AP flare; Fig. 5D). While 67 (38%) and 22 (28%) avapritinib- and midostaurin-treated patients, respectively, had an early decrease in serum AP levels ≥25% (range, –25 to –91%; –25 to –71%), 26 (15%) and 21 (27%) patients had neither increased nor decreased serum AP levels within 3 months after starting treatment. Median OS differed depending on the AP kinetics (avapritinib/midostaurin: AP flare, NR/99 months vs. decrease ≥25%, 52/42 months vs. others, 42/40 months; *p* = 0.0065/*p* = 0.185; Fig. 5E).

### Univariate and multivariable OS analyses of baseline and on-treatment parameters

The baseline mutation profiles *SRSF2/RUNX1/SETBP1* for avapritinib-treated patients and *SRSF2/ASXL1/EZH2* for midostaurin-treated patients were significant in univariate OS analyses (HR 0.293 [95% CI, 0.156–0.551] and 0.338 [95% CI, 0.180–0.635], respectively). In multivariable analysis, absence of mutations in *SRSF2/RUNX1/SETBP1* showed a trend (HR 0.500 [95% CI, 0.245–1.019]) and absence of mutations in *SRSF2/ASXL1/EZH2* remained associated with worse OS (HR 0.269 [95% CI, 0.112–0.647]).

Compared to absence of an AHN, presence of an AHN was associated with worse OS in avapritinib-treated (HR 5.35 [95% CI, 1.911−14.977]) and midostaurin-treated (HR 3.738 [95% CI, 1.468–9.565]) patients in univariate analyses only.

When considering the best treatment response to avapritinib achieved within the first 6 months, serum tryptase reduction ≥70% (HR 0.414 [95% CI, 0.216–0.793]), AP flare (HR 0.427 [95% CI, 0.224–0.814]), and *KIT* D816V VAF reduction ≥30% (HR 0.439 [95% CI, 0.204–0.943]) were significant in univariate analyses. The multivariable analysis revealed normalization of the hemoglobin and/or platelet counts (HR 0.275 [95% CI, 0.121–0.624]) and normalization of the albumin levels (HR 0.177 [95% CI, 0.062–0.506]) as significant factors predictive of OS (Supplementary Table S4).

When considering the best treatment response to midostaurin achieved within the first 6 months, BM MC reduction ≥25% (HR 0.430 [95% CI, 0.221–0.837]), serum tryptase reduction ≥25% (HR 0.427 [95% CI, 0.239–0.760]), and normalization of the albumin levels (HR 0.277 [95% CI, 0.140–0.545]) were significant in univariate analyses. The multivariable analysis revealed *KIT* D816V EAB reduction ≥25% (HR 0.377 [95% CI, 0.167–0.854]) and normalization of hemoglobin and/or platelet counts (HR 0.341 [95% CI, 0.136–0.858]) as significant factors predictive for OS (Supplementary Table S5).

Avapritinib rendered *KIT* D816V in PB undetectable in 24% of patients, whereas no such conversions were observed with midostaurin. Serum tryptase normalized to <11.4 ng/mL in 57% of avapritinib-treated patients compared with 10% of those receiving midostaurin. Normalization of hemoglobin (48%), platelets (49%), and albumin (88%) also occurred more frequently with avapritinib than with midostaurin (24%, 22%, and 65%, respectively). Similarly, although less pronounced, normalization rates favored avapritinib for leukocytes (94% vs. 81%), monocytes (89% vs. 78%), and eosinophils (96% vs. 91%) (Supplementary Table S6).

## Discussion

In AdvSM, midostaurin was approved by the US Food and Drug Administration (FDA) and the European Medicines Agency (EMA) on the basis of a single-arm, open-label, phase 2 trial (CPKC412D2201), which reported an overall response rate (ORR) of 60% according to modified Valent criteria [35]. In a post hoc analysis using more stringent International Working Group-Myeloproliferative Neoplasms Research and Treatment and European Competence Network on Mastocytosis (IWG-MRT-ECNM) criteria, the EMA and FDA modified the ORR to 28% and 17%, respectively. For avapritinib, the single-arm, open-label phase 1 EXPLORER trial and the single-arm, open-label, phase 2 PATHFINDER trial reported ORRs of 75% and 73%, respectively, by the modified IWG-MRT-ECNM criteria [40, 42]. Consequently, avapritinib was approved by the FDA in adults regardless of previous therapy and by the EMA as monotherapy in adult patients after ≥1 previous systemic therapies. The high ORR (87%) in the first-line setting was confirmed in the 4-year follow-up of PATHFINDER [43]. Cladribine has been widely used for nearly two decades because of efficacy reported in several case series, and it received orphan drug designation from the EMA in 2013 [22–26, 44–47].

Due to the rarity and heterogeneity of AdvSM, prospective randomized clinical trials including avapritinib, midostaurin, and/or cladribine are not anticipated. While retrospective comparisons are susceptible to inherent biases such as patient selection, treatment adherence, and inconsistencies in data documentation, they usually allow for longer follow-up than prospective studies. In two propensity-matched analyses, one reported superior OS for midostaurin over cladribine and the other reported superior OS for avapritinib over midostaurin and cladribine [21, 38].

The current analysis involved comprehensive assessment of clinical and genetic data at baseline and during follow-up in two large and independent cohorts. For both cohorts, we primarily confirmed the adverse impact of the *S/A/R* gene panel. Prognosis was even better predicted by mutations in *SRSF2/RUNX1/SETBP1* in avapritinib-treated patients and in *SRSF2/ASXL1/EZH2* in midostaurin-treated patients. A thorough analysis of *SRSF2* in avapritinib-treated patients revealed a predictable inferior OS in patients with the *SRSF2* P95 hotspot mutation, but unexpectedly also for patients with mutations in other regions of *SRSF2*.

Because response to treatment can abrogate a negative baseline risk categorization (e.g., a ≥25% reduction of the *KIT* D816V EAB on midostaurin at month 6 [mutation-adjusted risk score v2.0]) [20], we also investigated the impact of changes in various genetic, serologic, and morphologic parameters. The rate and extent of biomarker changes are of importance in AdvSM because the goals of treatment with targeted therapies include improved survival and reduction of disease burden. This is in contrast to indolent systemic mastocytosis, in which management focuses on symptom improvement.

In avapritinib-treated patients, the prognostic utility of monitoring the *KIT* D816V VAF was evident by a ≥10% reduction of the *KIT* D816V VAF as early as day 15. Predictive responses to avapritinib and/or midostaurin were observed in the form of normalization of hemoglobin and platelet counts and restoration of albumin levels. These findings support established response criteria such as the modified IWG-MRT-ECNM, which are based on improvement of C-findings. They also reinforce the value of the Mutation-Adjusted Risk Score (MARS), which incorporates age, mutations in *SRSF2/ASXL1/RUNX1*, hemoglobin levels, and platelet counts. While age and mutation status remain largely unchanged during treatment, hemoglobin and platelet counts are dynamic parameters and may improve with effective therapy. Albumin normalization, which is not part of MARS, may therefore provide additional prognostic information on treatment. In contrast to midostaurin, reduction of *KIT* D816V in avapritinib-treated patients did not remain significant in the multivariable model, likely because avapritinib induces far deeper reductions, even rendering *KIT* D816V undetectable in approximately one-quarter of patients, thereby limiting its discriminatory value.

An elevated AP is established as a diagnostic C-finding by the WHO/ICC [1, 2], as a prognostic marker within several risk scores [10, 17–19, 48], and as a response parameter by IWG-MRT-ECNM [49–51]. Alkaline phosphatase isoform analyses showed that elevated AP suggests liver involvement/damage by MC infiltration rather than bone involvement (e.g., osteoporosis, osteosclerosis, or osteolysis) [52]. In addition to absolute increase or decrease over time, we have described the prognostic utility of AP kinetics. In avapritinib-treated patients, an early AP flare within 3 months after start of treatment was associated with improved OS. In the absence of mechanistic data (e.g., paired liver biopsies were not available), we speculate that this transient AP flare may reflect treatment-induced MC depletion within the hepatic microenvironment, accompanied by MC mediator release and a localized inflammatory response, rather than disease progression or drug toxicity.

This study has several limitations inherent to the use of 2 independently assembled cohorts. The midostaurin cohort was derived from a real-world registry (GREM), whereas the avapritinib cohort originated from prospective clinical trials (EXPLORER/PATHFINDER), with anticipated differences in eligibility criteria, follow-up schedules, treatment adherence, and completeness of longitudinal data capture. In addition, genetic assessments were not harmonized across cohorts (different *KIT* D816V assays and nonidentical next-generation sequencing panels), which limits direct comparisons of mutation frequencies and allele burden reductions. The proposed genetic signatures for midostaurin (*SRSF2/ASXL1/EZH2*) and avapritinib (*SRSF2/RUNX1/SETBP1*) were derived from univariate analyses and were not cross-validated.

In conclusion, our data underscore the therapeutic paradigm of targeted KIT inhibition in patients with AdvSM and support the effectiveness of avapritinib as a relevant treatment option in this population. The intricate interplay of the multilineage involvement of *KIT* D816V and the contemporaneous presence of variable multimutated clones are the main contributors to the individual disease heterogeneity and the challenges for response assessment in AdvSM (e.g., the simultaneous presence and the frequently observed divergent response and progression of SM, AHN, or both compartments). We identified disparate genetic signatures conferring worse prognosis on avapritinib (*SRSF2/RUNX1/SETBP1*) and midostaurin (*SRSF2/ASXL1/EZH2*) and contextualized the utility of established and new on-treatment parameters on prognosis, such as AP flare. Our data may therefore help to identify situations where patients on midostaurin should be switched to avapritinib, or where patients on midostaurin or avapritinib might benefit from treatment intensification (e.g., high-dose chemotherapy with or without subsequent alloHCT). To further advance the treatment algorithms, future work should address simultaneous and sequential combinations (e.g., toward SM- and AHN-directed compartments) and to optimize the role and timing of KIT inhibition pre- and post-alloHCT [28].

## Supplementary information

Supplement Clean

## Data Availability

The datasets used and/or analyzed during the current study, including detailed variant-level information, are available from the corresponding author on reasonable request.
