# Supplementary material for: Predicting the clinical course of advanced systemic mastocytosis on treatment with avapritinib or midostaurin
Source: Leukemia. 2026 Oct 9;40(11):2562–72. doi: 10.1038/s41375-026-03149-0 (PMC13713545; doi:10.1038/s41375-026-03149-0)
Supplement: Supplementary file 1 [file 41375_2026_3149_MOESM1_ESM.docx]

**Supplementary Information**

**Predicting the clinical course of advanced systemic mastocytosis on treatment with avapritinib or midostaurin**

Johannes Lübke, Jason Gotlib, Daniel J. DeAngelo, Juliana Schwaab, Deepti H. Radia, Michael Deininger, Tracy I. George, Ilda Bidollari, Saša Dimitrijević, Javier I. Munoz-Gonzalez, Hongliang Shi and Andreas Reiter

**Table of contents**

**Supplementary
Text** ………………………………………………………………………………………………..….... 2

**Supplementary Tables**

**Table S1:** Baseline disease characteristics by *KIT* D816V allele burden reduction at cycle 1,
day 5 ………..……………………………………………………………………………………..…… 3

**Table S2:** Baseline disease characteristics by serum tryptase reduction at cycle 3, day 1…... 5

**Table S3:** Baseline disease characteristics by serum tryptase halving time within 30 days …. 7

**Table S4:** Univariate and multivariable analyses for avapritinib ………….……………..………. 9

**Table S5:** Univariate and multivariable analyses for midostaurin .…....……………………….. 10

**Table S6:** Normalization of on-treatment parameters by treatment .…………………………... 11

**Supplementary Figures**

**Fig. S1:** Genomic landscape of Tier 1 additional somatic mutations beyond the canonical driver mutation *KIT* D816V (and other rare D816 variants) in patients with AdvSM treated
with avapritinib ……………………………………………………………………………………..... 12

**Fig. S2:** Genomic landscape of additional somatic mutations beyond the canonical driver mutation *KIT* D816V (and other rare D816 variants) in patients with AdvSM treated
with midostaurin ….……..…………………………………………………………………...………. 13

**Fig. S3:** Variant allele frequencies across different somatic mutations ..….…………….…….. 14

**Fig. S4:** Overall survival in *SRSF2*-mutated patients with AdvSM ..………….…….….….…… 15

**Data collection**

*Avapritinib.* Efficacy and safety of avapritinib in patients with advanced systemic mastocytosis were studied in the phase 1 EXPLORER (NCT02561988) and phase 2 PATHFINDER (NCT03580655) trials. Data from patients treated with avapritinib in both trials were pooled for this analysis.

*Midostaurin.* The German Registry on Eosinophils and Mast Cells (GREM) originated in 2012 from the Registry for Rare Myeloproliferative Disorders. The GREM is coordinated by the Medical Faculty Mannheim (Heidelberg University), which is considered a center of excellence by the European Competence Network on Mastocytosis.

**Myeloid next-generation-sequencing panel**

*Avapritinib*. A TruSight™ Myeloid NGS Panel was used, and the following 54 genes were analyzed: *ABL1, ASXL1, ATRX, BCOR, BCORL1, BRAF, CALR, CBL, CBLB, CBLC, CDKN2A, CEBPA, CSF3R, CUX1, DNMT3A, ETV6/TEL, EZH2, FBXW7, FLT3, GATA1, GATA2, GNAS, HRAS, IDH1, IDH2, IKZF1, JAK2, JAK3, KDM6A, KIT, KRAS, MLL, MPL, MYD88, NOTCH1, NPM1, NRAS, PDGFRA, PHF6, PTEN, PTPN11, RAD21, RUNX1, SETBP1, SF3B1, SMC1A, SMC3, SRSF2, STAG2, TET2, TP53, U2AF1, WT1,* and *ZRSR2.*

*Midostaurin*. The following 33 genes were analyzed: *ASXL1, BCOR, CALR, CBL, CSNK1A1, DNMT3A, ETNK1, ETV6, EZH2, FLT3, GATA1, GATA2, IDH1, IDH2, JAK2, KIT, KRAS, MLL, MPL, NPM1, NRAS, PHF6, PIGA, PTPN11, RUNX1, SETBP1, SF3B1, SRSF2, TET2, TP53, U2AF1, ZRSR2,* and *WT1*.

| **Table S1:** Baseline disease characteristics by *KIT* D816V allele burden reduction at cycle 1, day 15. | | | | | | | |
| --- | --- | --- | --- | --- | --- | --- | --- |
|  | | |  | **≥10%** |  | **<10%** |  |
| Number of patients | | |  | 86 |  | 58 |  |
| Age in years at treatment initiation, median (range) | | |  | 69 (31–83) |  | 65 (43–88) |  |
| Male, *n* (%) | | |  | 51 (59) |  | 32 (55) |  |
|  | | |  |  |  |  |  |
| Diagnosis | | |  |  |  |  |  |
|  | ASM, *n* (%) | |  | 14 (16) |  | 10 (17) |  |
|  | SM-AHN, *n* (%) | |  | 58 (67) |  | 43 (74) |  |
|  | MCL±AHN, *n* (%) | |  | 14 (16) |  | 5 (9) |  |
|  |  | |  |  |  |  |  |
| AHN | | |  |  |  |  |  |
|  | CMML, *n* (%) | |  | 30 (35) |  | 18 (31) |  |
|  | MDS/MPN, *n* (%) | |  | 15 (17) |  | 11 (19) |  |
|  | MDS, *n* (%) | |  | 7 (8) |  | 8 (14) |  |
|  | CEL, *n* (%) | |  | 3 (3) |  | 3 (5) |  |
|  | Others, *n* (%) | |  | 4 (5) |  | 4 (7) |  |
|  |  | |  |  |  |  |  |
| Relevant laboratory and clinical parameters for the assessment of C-findings | | | | | | |  |
|  | Hemoglobin, g/dL, median (range) | |  | 10 (7–14) |  | 11 (8–17) |  |
|  |  | <10 g/dL, *n* (%) |  | 36 (42) |  | 15 (26) |  |
|  | Platelets, × 10^9^/L, median (range) | |  | 147 (28–992) |  | 154 (28–606) |  |
|  |  | <100 × 10^9^/L, *n* (%) |  | 26 (30) |  | 23 (40) |  |
|  | ANC, × 10^9^/L, median (range) | |  | 5 (0–32) |  | 5 (0–44) |  |
|  |  | <1 × 10^9^/L, *n* (%) |  | 5 (6) |  | 3 (5) |  |
|  | AP, U/L, median (range) | |  | 153 (53–1 150) |  | 191 (33–1 747) |  |
|  |  | >150 U/L, *n* (%) |  | 44 (51) |  | 33 (57) |  |
|  | Albumin level, g/L, median (range) | |  | 40 (13–49) |  | 39 (23–50) |  |
|  |  | <34 g/L, *n* (%) |  | 17 (20) |  | 11 (19) |  |
|  | Weight loss (>10% over 6 months), *n* (%) | |  | 17 (20) |  | 17 (29) |  |
|  | | |  |  |  |  |  |
| Other relevant parameters | | |  |  |  |  |  |
|  | Leukocytes, × 10^9^/L, median (range) | |  | 9 (1–45) |  | 8 (1–45) |  |
|  | Monocytes, × 10^9^/L, median (range) | |  | 1 (0–10) |  | 1 (0–7) |  |
|  | Eosinophils, × 10^9^/L, median (range) | |  | 0 (0–9) |  | 0 (0–24) |  |
|  | MC infiltration in BM, %, median (range) | |  | 40 (5–95) |  | 50 (1–95) |  |
|  | Serum tryptase level, µg/L, median (range) | |  | 300 (12–1 600) |  | 173 (37–772) |  |
|  | *KIT* D816V EAB/VAF in PB, %, median (range) | |  | 20 (0–80) |  | 19 (0–49) |  |
|  |  | |  |  |  |  |  |
| MARS at diagnosis | | |  |  |  |  |  |
|  | Low risk, *n* (%) | |  | 26 (30) |  | 23 (40) |  |
|  | Intermediate risk, *n* (%) | |  | 22 (26) |  | 11 (19) |  |
|  | High risk, *n* (%) | |  | 38 (44) |  | 24 (41) |  |
|  |  | |  |  |  |  |  |
| Prior treatment | | |  |  |  |  |  |
|  | Cladribine, *n* (%) | |  | 6 (7) |  | 6 (10) |  |
|  | Midostaurin, *n* (%) | |  | 39 (45) |  | 19 (33) |  |
| **Abbreviations:** *AHN* associated hematologic neoplasm, *ANC* absolute neutrophil count, *AP* alkaline phosphatase, *ASM* aggressive systemic mastocytosis, *BM* bone marrow, *CEL* chronic eosinophilic leukemia, *CMML* chronic myelomonocytic leukemia, *EAB* expressed allele burden, *MARS* mutation-adjusted risk score, *MC* mast cell, *MCL±AHN* mast cell leukemia with/without an associated hematologic neoplasm, *MDS* myelodysplastic syndrome, *MDS/MPN* myelodysplastic syndrome/myeloproliferative neoplasm, *PB* peripheral blood, *SM-AHN* systemic mastocytosis with an associated hematologic neoplasm, *VAF* variant allele frequency. | | | | | | | |

| **Table S2:** Baseline disease characteristics by serum tryptase reduction at cycle 3, day 1. | | | | | | | |
| --- | --- | --- | --- | --- | --- | --- | --- |
|  | | |  | **≥60%** |  | **<60%** |  |
| Number of patients | | |  | 124 |  | 32 |  |
| Age in years at treatment initiation, median (range) | | |  | 68 (31-88) |  | 69 (37-83) |  |
| Male, *n* (%) | | |  | 72 (58) |  | 18 (56) |  |
|  | | |  |  |  |  |  |
| Diagnosis | | |  |  |  |  |  |
|  | ASM, *n* (%) | |  | 23 (19) |  | 2 (6) |  |
|  | SM-AHN, *n* (%) | |  | 81 (65) |  | 25 (78) |  |
|  | MCL±AHN, *n* (%) | |  | 20 (16) |  | 5 (16) |  |
|  |  | |  |  |  |  |  |
| AHN | | |  |  |  |  |  |
|  | CMML, *n* (%) | |  | 35 (28) |  | 14 (44) |  |
|  | MDS/MPN, *n* (%) | |  | 23 (19) |  | 7 (22) |  |
|  | MDS, *n* (%) | |  | 10 (8) |  | 6 (19) |  |
|  | CEL, *n* (%) | |  | 6 (5) |  | 0 |  |
|  | Others, *n* (%) | |  | 8 (6) |  | 0 |  |
|  |  | |  |  |  |  |  |
| Relevant laboratory and clinical parameters for the assessment of C-findings | | | | | | |  |
|  | Hemoglobin, g/dL, median (range) | |  | 11 (7–17) |  | 10 (7–14) |  |
|  |  | <10 g/dL, *n* (%) |  | 41 (33) |  | 16 (50) |  |
|  | Platelets, × 10^9^/L, median (range) | |  | 150 (28–992) |  | 118 (28–382) |  |
|  |  | <100 × 10^9^/L, *n* (%) |  | 37 (30) |  | 14 (44) |  |
|  | ANC, × 10^9^/L, median (range) | |  | 5 (0–32) |  | 5 (1–27) |  |
|  |  | <1 × 10^9^/L, *n* (%) |  | 7 (6) |  | 0 |  |
|  | AP, U/L, median (range) | |  | 157 (33–1 747) |  | 204 (27–811) |  |
|  |  | >150 U/L, *n* (%) |  | 66 (53) |  | 19 (59) |  |
|  | Albumin level, g/L, median (range) | |  | 40 (13–50) |  | 38 (28–46) |  |
|  |  | <34 g/L, *n* (%) |  | 21 (17) |  | 10 (31) |  |
|  | Weight loss (>10% over 6 months), *n* (%) | |  | 27 (22) |  | 7 (22) |  |
|  | | |  |  |  |  |  |
| Other relevant parameters | | |  |  |  |  |  |
|  | Leukocytes, × 10^9^/L, median (range) | |  | 9 (1–42) |  | 9 (2–45) |  |
|  | Monocytes, × 10^9^/L, median (range) | |  | 1 (0–10) |  | 1 (0–8) |  |
|  | Eosinophils, × 10^9^/L, median (range) | |  | 0 (0–24) |  | 0 (0–5) |  |
|  | MC infiltration in BM, %, median (range) | |  | 50 (10–95) |  | 45 (5–90) |  |
|  | Serum tryptase level, µg/L, median (range) | |  | 234 (24–1 600) |  | 172 (21–1 600) |  |
|  | *KIT* D816V EAB/VAF in PB, %, median (range) | |  | 15 (0–49) |  | 20 (0–80) |  |
|  |  | |  |  |  |  |  |
| MARS at diagnosis | | |  |  |  |  |  |
|  | Low risk, *n* (%) | |  | 50 (40) |  | 7 (22) |  |
|  | Intermediate risk, *n* (%) | |  | 28 (23) |  | 6 (19) |  |
|  | High risk, *n* (%) | |  | 46 (37) |  | 19 (59) |  |
|  |  | |  |  |  |  |  |
| Prior treatment | | |  |  |  |  |  |
|  | Cladribine, *n* (%) | |  | 7 (6) |  | 5 (16) |  |
|  | Midostaurin, *n* (%) | |  | 45 (36) |  | 13 (41) |  |
| **Abbreviations:** *AHN* associated hematologic neoplasm*, ANC* absolute neutrophil count, *AP* alkaline phosphatase, *ASM* aggressive systemic mastocytosis, *BM* bone marrow, *CEL* chronic eosinophilic leukemia, *CMML* chronic myelomonocytic leukemia, *EAB* expressed allele burden, *MARS* mutation-adjusted risk score, *MC* mast cell, *MCL±AHN* mast cell leukemia with/without an associated hematologic neoplasm, *MDS* myelodysplastic syndrome, *MDS/MPN* myelodysplastic syndrome/myeloproliferative neoplasm, *PB* peripheral blood, *SM-AHN* systemic mastocytosis with an associated hematologic neoplasm, *VAF* variant allele frequency. | | | | | | | |

| **Table S3:** Baseline disease characteristics by serum tryptase halving time within 30 days. | | | | | | | |
| --- | --- | --- | --- | --- | --- | --- | --- |
|  | | |  | **≤30 days** |  | **>30 days** |  |
| Number of patients | | |  | 145 |  | 23 |  |
| Age in years at treatment initiation, median (range) | | |  | 68 (31–88) |  | 73 (54–86) |  |
| Male, *n* (%) | | |  | 83 (57) |  | 15 (65) |  |
|  | | |  |  |  |  |  |
| Diagnosis | | |  |  |  |  |  |
|  | ASM, *n* (%) | |  | 29 (20) |  | 0 |  |
|  | SM-AHN, *n* (%) | |  | 89 (61) |  | 23 (100) |  |
|  | MCL±AHN, *n* (%) | |  | 27 (19) |  | 0 |  |
|  |  | |  |  |  |  |  |
| AHN | | |  |  |  |  |  |
|  | CMML, *n* (%) | |  | 40 (28) |  | 16 (70) |  |
|  | MDS/MPN, *n* (%) | |  | 26 (18) |  | 2 (9) |  |
|  | MDS, *n* (%) | |  | 11 (8) |  | 4 (17) |  |
|  | CEL, *n* (%) | |  | 6 (4) |  | 0 |  |
|  | Others, *n* (%) | |  | 9 (6) |  | 1 (4) |  |
|  |  | |  |  |  |  |  |
| Relevant laboratory and clinical parameters for the assessment of C-findings | | | | | | |  |
|  | Hemoglobin, g/dL, median (range) | |  | 11 (7–17) |  | 10 (7–13) |  |
|  |  | <10 g/dL, *n* (%) |  | 49 (34) |  | 12 (52) |  |
|  | Platelets, × 10^9^/L, median (range) | |  | 152 (28–992) |  | 94 (49–382) |  |
|  |  | <100 × 10^9^/L, *n* (%) |  | 44 (30) |  | 12 (52) |  |
|  | ANC, × 10^9^/L, median (range) | |  | 4 (0–32) |  | 6 (0–27) |  |
|  |  | <1 × 10^9^/L, *n* (%) |  | 7 (5) |  | 1 (4) |  |
|  | AP, U/L, median (range) | |  | 151 (27–1 747) |  | 257 (92–811) |  |
|  |  | >150 U/L, *n* (%) |  | 73 (50) |  | 16 (70) |  |
|  | Albumin level, g/L, median (range) | |  | 40 (13–50) |  | 36 (28–47) |  |
|  |  | <34 g/L, *n* (%) |  | 24 (17) |  | 8 (35) |  |
|  | Weight loss (>10% over 6 months), *n* (%) | |  | 31 (21) |  | 6 (26) |  |
|  | | |  |  |  |  |  |
| Other relevant parameters | | |  |  |  |  |  |
|  | Leukocytes, × 10^9^/L, median (range) | |  | 9 (1–43) |  | 11 (2–45) |  |
|  | Monocytes, × 10^9^/L, median (range) | |  | 1 (0–9) |  | 2 (0–10) |  |
|  | Eosinophils, × 10^9^/L, median (range) | |  | 0 (0–24) |  | 0 (0–5) |  |
|  | MC infiltration in BM, %, median (range) | |  | 50 (3–95) |  | 30 (5–80) |  |
|  | Serum tryptase level, µg/L, median (range) | |  | 238 (12–1 600) |  | 137 (21–856) |  |
|  | *KIT* D816V EAB/VAF in PB, %, median (range) | |  | 13 (0–49) |  | 20 (1–80) |  |
|  |  | |  |  |  |  |  |
| MARS at diagnosis | | |  |  |  |  |  |
|  | Low risk, *n* (%) | |  | 58 (40) |  | 3 (13) |  |
|  | Intermediate risk, *n* (%) | |  | 31 (21) |  | 4 (17) |  |
|  | High risk, *n* (%) | |  | 56 (39) |  | 16 (70) |  |
|  |  | |  |  |  |  |  |
| Prior treatment | | |  |  |  |  |  |
|  | Cladribine, *n* (%) | |  | 8 (6) |  | 4 (17) |  |
|  | Midostaurin, *n* (%) | |  | 47 (32) |  | 11 (48) |  |
| **Abbreviations:** *AHN* associated hematologic neoplasm, *ANC* absolute neutrophil count, AP alkaline phosphatase, *ASM* aggressive systemic mastocytosis, *BM* bone marrow, *CEL* chronic eosinophilic leukemia, *CMML* chronic myelomonocytic leukemia, *EAB* expressed allele burden, *MARS* mutation-adjusted risk score, *MC* mast cell, *MCL±AHN* mast cell leukemia with/without an associated hematologic neoplasm, *MDS* myelodysplastic syndrome, *MDS/MPN* myelodysplastic syndrome/myeloproliferative neoplasm, *PB* peripheral blood, *SM-AHN* systemic mastocytosis with an associated hematologic neoplasm, *VAF* variant allele frequency. | | | | | | | |

| **Table S4:** Univariate and multivariable analyses for avapritinib. | | | | | | | |
| --- | --- | --- | --- | --- | --- | --- | --- |
|  | **Univariate analysis** | | |  | **Multivariable analysis^a^** | | |
| Characteristics:  best response within  6 months^b^ |  | HR (95% CI) | *p* |  |  | HR (95% CI) | *p* |
| Mast cells in BM section (%):  reduction <50 (ref) vs. ≥50^c^ |  | 0.477 (0.218–1.042) | 0.063 |  |  |  |  |
| Serum tryptase (%):  reduction <70 (ref) vs. ≥70^d^ |  | **0.414 (0.216–0.793)** | **0.008** |  |  | 0.680 (0.338–1.369) | 0.280 |
| AP flare:  no (ref) vs. yes |  | **0.427 (0.224–0.814)** | **0.010** |  |  | 0.667 (0.322–1.382) | 0.276 |
| Hemoglobin/platelets normalization:  no (ref) vs. yes |  | **0.210 (0.100–0.439)** | **<0.001** |  |  | **0.275 (0.121–0.624)** | **0.002** |
| Albumin normalization:  no (ref) vs. yes |  | **0.317 (0.124–0.810)** | **0.017** |  |  | **0.177 (0.062–0.506)** | **0.001** |
| Leukocytes (%):  reduction <50 (ref) vs. ≥50^d^ |  | 1.952 (0.769–4.951) | 0.159 |  |  |  |  |
| Monocytes (%):  reduction <10 (ref) vs. ≥10^d^ |  | 0.533 (0.128–2.212) | 0.386 |  |  |  |  |
| Eosinophils (%):  reduction <75 (ref) vs. ≥75^d^ |  | 3.198 (0.991–10.323) | 0.052 |  |  |  |  |
| *KIT* response in PB (%):  reduction <30 (ref) vs. ≥30^e^ |  | **0.439 (0.204–0.943)** | **0.035** |  |  | 0.549 (0.239–1.261) | 0.158 |
| *SRSF2/RUNX1/SETBP1*:  pos (ref) vs. neg |  | **0.293 (0.156–0.551)** | **<0.001** |  |  | 0.500 (0.245–1.019) | 0.057 |
| AHN^f^:  Absence (ref) vs. presence |  | **5.350 (1.911–14.977)** | **0.001** |  |  | 1.916 (0.631–5.814) | 0.251 |
| ^a^The multivariable model was adjusted by incorporating the following baseline parameters (significant in the univariate analyses): age, mutation status in *SRSF2/RUNX1/SETBP1*. ^b^Except for AP flare (3 months). ^c^Or resolution. ^d^Or normalization. ^e^*KIT* D816V was sequentially measured on DNA basis. ^f^The presence or absence of AHN was assessed across different advanced systemic mastocytosis subgroups, including patients with mast cell leukemia. HR >1 indicates worse overall survival compared with the reference group (absence of AHN)  **Abbreviations:** *AHN* associated hematologic neoplasm, *AP* alkaline phosphatase, *BM* bone marrow, *CI* confidence interval, *HR* hazard ratio, *n* evaluable number of patients, *neg* negative, *PB* peripheral blood, *pos* positive. | | | | | | | |

| **Table S5:** Univariate and multivariable analyses for midostaurin. | | | | | | | |
| --- | --- | --- | --- | --- | --- | --- | --- |
|  | **Univariate analysis** | | |  | **Multivariable analysis^a^** | | |
| Characteristics:  best response within  6 months^b^ |  | HR (95% CI) | *p* |  |  | HR (95% CI) | *p* |
| Mast cells in BM section (%):  reduction <25 (ref) vs. ≥25^c^ |  | **0.430 (0.221**–**0.837)** | **0.013** |  |  | 0.546 (0.230–1.297) | 0.171 |
| Serum tryptase (%):  reduction <25 (ref) vs. ≥25^d^ |  | **0.427 (0.239**–**0.760)** | **0.004** |  |  | 0.660 (0.306–1.424) | 0.290 |
| AP flare:  no (ref) vs. pos |  | 0.560 (0.312–1.004) | 0.051 |  |  |  |  |
| Albumin normalization:  no (ref) vs. pos |  | **0.277 (0.140**–**0.545)** | **<0.001** |  |  | 0.643 (0.271–1.528) | 0.318 |
| Hemoglobin/platelets normalization:  no (ref) vs. pos |  | **0.275 (0.146**–**0.519)** | **<0.001** |  |  | **0.341 (0.136**–**0.858)** | **0.022** |
| Leukocytes (%):  reduction <80 (ref) vs. ≥80^d^ |  | 0.392 (0.139–1.109) | 0.078 |  |  |  |  |
| Monocytes (%):  reduction <20 (ref) vs. ≥20^d^ |  | 1.171 (0.654–2.100) | 0.595 |  |  |  |  |
| Eosinophils (%):  reduction <20 (ref) vs. ≥20^d^ |  | 0.480 (0.189–1.221) | 0.123 |  |  |  |  |
| *KIT* response in PB (%):  reduction <25 (ref) vs. ≥25^e^ |  | 0.386 (0.195–0.765) | **0.006** |  |  | **0.377 (0.167**–**0.854)** | **0.019** |
| *SRSF2/ASXL1/EZH2*:  pos (ref) vs. neg |  | **0.338 (0.180**–**0.635)** | **<0.001** |  |  | **0.269 (0.112**–**0.647)** | **0.003** |
| AHN:  presence (ref) vs. absence^f^ |  | **3.738 (1.468**–**9.517)** | **0.006** |  |  | 2.094 (0.459–9.565) | 0.340 |
| ^a^The multivariable model was adjusted by incorporating the following baseline parameters (significant in the univariate analyses): age, leukocyte count, eosinophil count, mutation status in *SRSF2/ASXL1/EZH2*. ^b^Except for AP flare (3 months). ^c^Or resolution. ^d^Or normalization. ^e^*KIT* D816V was sequentially measured on RNA basis. ^f^The presence or absence of AHN was assessed across different advanced systemic mastocytosis subgroups, including patients with mast cell leukemia.  **Abbreviations:** *AHN* associated hematologic neoplasm, *AP* alkaline phosphatase, *BM* bone marrow, *CI* confidence interval, *HR* hazard ratio, *n* evaluable number of patients, *neg* negative, *PB* peripheral blood, *pos* positive. | | | | | | | |

| **Table S6:** Normalization of on-treatment parameters by treatment. | | |
| --- | --- | --- |
| **On-treatment parameters** | **Avapritinib, *n/N* (%)** | **Midostaurin, *n/N* (%)** |
| Serum tryptase (baseline ≥11.4 to post-baseline <11.4 ng/mL) | 99/175 (57) | 8/79 (10) |
| Hemoglobin (baseline <LLN to post-baseline within normal range) | 64/133 (48) | 11/46 (24) |
| Platelets (baseline <LLN to post-baseline within normal range) | 41/84 (49) | 11/51 (22) |
| Albumin (baseline <LLN to post-baseline within normal range) | 36/41 (88) | 22/34 (65) |
| Leukocytes (baseline >ULN to post-baseline within normal range) | 68/72 (94) | 30/37 (81) |
| Monocytes (baseline >ULN to post-baseline within normal range) | 78/88 (89) | 28/36 (78) |
| Eosinophils (baseline >ULN to post-baseline within normal range) | 46/48 (96) | 29/32 (91) |
| *KIT* D816V EAB/VAF in PB (baseline detectable to post-baseline not detectable) | 39/160 (24) | 0/74 (0) |
| **Abbreviations:** *EAB* expressed allele burden, *LLN* lower limit of normal, *PB* peripheral blood, *ULN* upper limit of normal, *VAF* variant allele frequency | | |

**Supplementary Fig. S1	Genomic landscape of Tier 1 additional somatic mutations beyond the canonical driver mutation *KIT* D816V (and other rare D816 variants) in patients with AdvSM treated with avapritinib.** Panel **A** shows the frequency of Tier 1 mutations in 176 patients with AdvSM treated with avapritinib. Mutations were categorized by type (missense, in-frame deletions or insertions [Inframe indel], frameshift deletions or insertions, whether they resulted in a truncation or were affecting a splice site). Colors represent the type of location. In Panel **B**, the Circos diagram highlights the genetic background of AdvSM. The links between different genetic lesions are represented using various colors, with directional arrows indicating their relationships. The width of the links reflects the strength of these relationships. The sector widths signify the overall strength of a genetic lesion to connect or be connected to other genetic lesions. Panel **C** presents the frequency of additional somatic mutations beyond *KIT*. **Abbreviations:** *add* additional, *AdvSM* advanced systemic mastocytosis, *mut* mutation.

**Supplementary Fig. S2	Genomic landscape of additional somatic mutations beyond the canonical driver mutation *KIT* D816V (and other rare D816 variants) in patients with AdvSM treated with midostaurin.** Panel **A** shows the frequency mutations in 79 midostaurin-treated patients with AdvSM. Mutations were categorized by type (missense, in-frame deletions or insertions [Inframe indel], frameshift deletions or insertions, or whether they resulted in a truncation). Colors represent the type of location. The asterisks indicate whether the presence/absence of a genetic lesion was associated with overall survival. Multiple asterisks correspond to higher hazard ratios. In Panel **B**, the links between different genetic lesions are represented using various colors, with directional arrows indicating the relationships. The width of the links reflects the strength of these relationships. The sector widths signify the overall strength of a genetic lesion to connect or be connected to other genetic lesions. Panel **C** presents the number of mutated genes per patients. **Abbreviation:** *AdvSM* advanced systemic mastocytosis, *mut* mutation.

**Supplementary Fig. S3	Variant allele frequencies across different somatic mutations.** Raincloud plots visualize the distribution of VAFs of additional somatic mutations, each with an individual prevalence of at least 15% within the population of avapritinib-treated patients with advanced systemic mastocytosis. **Abbreviation:** *VAF* variant allele frequency.

**Supplementary Fig. S4	Overall survival in *SRSF2*-mutated patients with AdvSM.** Kaplan–Meier probability estimates of OS in avapritinib-treated patients with AdvSM based on different P95 hotspot mutations. **Abbreviations:** *AdvSM* advanced systemic mastocytosis, *OS* overall survival.
